# Biomimetic Yeast Cell Typing—Application of QCMs

**DOI:** 10.3390/s91008146

**Published:** 2009-10-16

**Authors:** Karin Seidler, Miroslava Polreichová, Peter A. Lieberzeit, Franz L. Dickert

**Affiliations:** University of Vienna, Department of Analytical Chemistry and Food Chemistry, Faculty of Chemistry, Waehringer Strasse 38, A-1090 Vienna, Austria; E-Mails: karin.seidler@univie.ac.at (K.S.); miroslava.polreichova@univie.ac.at (M.P.); peter.lieberzeit@univie.ac.at (P.A.L.)

**Keywords:** molecular imprinting, yeast, QCM, bioanalyte sensing, process control

## Abstract

Artificial antibodies represent a key factor in the generation of sensing systems for the selective detection of bioanalytes of variable sizes. With biomimetic surfaces, the important model organism *Saccharomyces cerevisiae* and several of its growth stages may be detected. Quartz crystal microbalances (QCM) with 10 MHz fundamental frequency and coated with polymers imprinted with synchronized yeast cells are presented, which are able to detect duplex cells with high selectivity. Furthermore, a multichannel quartz crystal microbalance (MQCM) was designed and optimized for the measurement in liquids. This one-chip system based on four-electrode geometry allows the simultaneous detection of four analytes and, thus, provides a monitoring system for biotechnology and process control. For further standardization of the method, synthetic stamps containing plastic yeast cells in different growth stages were produced and utilized for imprinting. Mass-sensitive measurements with such MIPs resulted in the same sensor characteristics as obtained for those imprinted with native yeast cells.

## 1. Introduction

Over the last decade, label-free detection of bioanalytes has become a center of focus in analytical chemistry. The development of reliable sensing systems for biological species and molecules is of substantial interest not only for medicine or fundamental research but also for the application in biotechnology and process control. Combining molecularly imprinted polymers with mass-sensitive devices provides robust and miniaturizable measuring systems, which have proven to be highly suitable for this purpose. Due to their easy adaptability to a variety of analytes/templates ranging from a few nm to several μm, MIPs are used versatilely—e.g., for the detection of blood, insulin, cells, viruses or even immunoglobulins [1,2]. In this paper, we have focused on the selective identification of the significant model organism *S. cerevisiae* in different stages of its cell cycle. Ten MHz QCMs were coated with polyurethane imprinted with a monolayer of duplex yeast cells. These were extracted by treating the cell culture with *N*-hydroxyurea, which blocks the cell cycle at the end of the synthesis phase due to inhibition of DNA replication. The resulting MIPs proved to be not only highly sensitive in the detection of synchronized yeast cells but also to be selective, since the cross-sensitivity to yeasts in their single cell stage is comparatively low [3,4].

Another task in the development of reliable sensor systems is improving reproducibility and standardizing the manufacturing process. Thus, we placed the focus on facilitating and optimizing the imprinting procedure by generating artificial master stamps of the template used. Stamp imprinting with such plastic cells can outperform their native analogues, since the corresponding cavities are less dependent on the individual cell properties of the respective stamp used. Here, synthetic copies of single and growing yeast cells are introduced and the resulting sensor characteristics are presented.

Chemical sensors based on quartz crystal microbalances are characterized by the respective transducer properties. Important features like sensitivity, reproducibility and accuracy of the frequency response strongly depend on the morphology of both, the crystal surface and the coating. In order to minimize these effects, a different strategy is presented in this work. So-called multichannel quartz crystal microbalances (MQCM) have been studied with increasing interest in recent years [5,6] and present a promising approach for various biosensor applications [7]. Here, we designed and tested QCM with 4-electrode geometry to employ them in liquid phase. One of the major problems in design is to minimize the crosstalk between adjacent channels. Successful solutions to circumvent this matter comprise the proper adjustment of electrode size or the utilization of mesa structures [8]. Decreasing the thickness of the gold electrodes and increasing the interchannel spacing between them also results in reduced interference, since the thickness shear wave decays exponentially outside the electrodes [9]. However, in this paper we present an effective measuring system based on a 4-electrode QCM and adapted to measuring in liquid phase. Surface imprinting with different growth stages of *S. cerevisiae* finally resulted in a biomimetic chemosensor suitable to monitor the growth development of this important microorganism.

## 2. Results and Discussion

### 2.1. Studying Cell Stages

**Figure 1.**
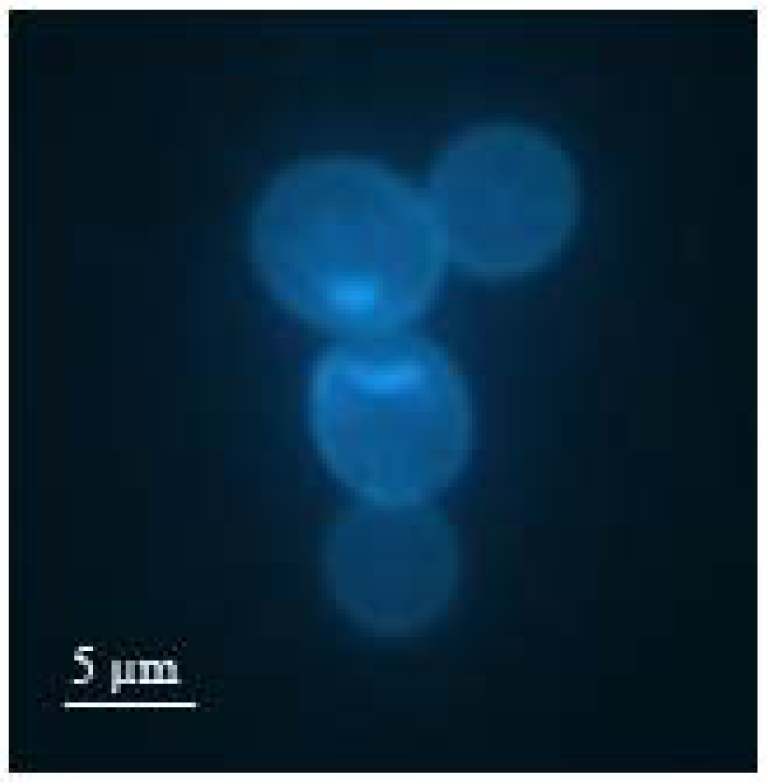
Budding yeast (S. cerevisiae) in late S phase marked with the fluorochrome bisbenzimide (fluorescence microscopy).

In molecular imprinting, surface cavities are formed by optimal alignment of the pre-polymer backbone around the template. This results in optimized fit between the final layer and the respective analyte both with respect to geometry and steric functionality. As yeast undergoes different growth stages during its reproductive cycle, the size and shape of the cell changes, whereas the chemical properties on its surface remain basically constant. Yet, by mass-sensitive detection with QCM, individual growth stages can be distinguished. According to Sauerbrey, the mass increase on the MIP-surface leads to a linear frequency decline of the oscillating quartz crystal. Such gravimetric sensors able to detect different stages in the development of yeast are reliable and easy to operate. Hence, they could be applied in biotechnology and in industrial fermentation processes, thus opening up new opportunities for monitoring microorganisms on-line. For the imprinting procedure with synchronized yeast, the cell culture was treated with *N*-hydroxyurea, which stops the growth at the end of S (or synthesis) phase. Due to inhibition of DNA replication after budding, cell division is prevented. At this point of growth, the eukaryote exists in a duplex form in the size of approximately 9 μm, which is slightly smaller than two single cells. Figure 1 shows a fluorescence microscope image of such a duplex cell marked with bisbenzimide. Here, it can be observed that the bud has reached roughly the size of the mother cell.

**Figure 2.**
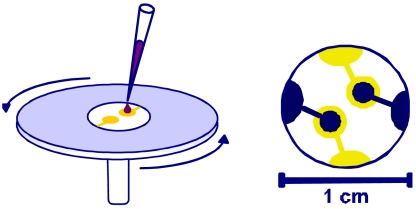
Imprinting procedure with synchronized yeast: cell suspension is dropped on PU-layer and spun at 2,500 rpm.

In order to generate optimum cavities in the polymer, the duplex cell suspension was dripped onto the polyurethane coating and immediately spin cast allowing the cells to arrange in horizontal position (Figure 2).

**Figure 3.**
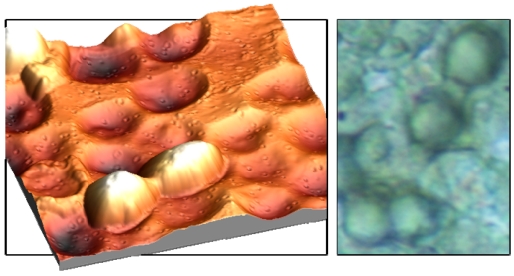
AFM image (lateral dimensions 20 × 20 μm, vertical axis 5 μm) of duplex yeast cell in MIP and imprints of duplex cells (light microscopy, 20 × 25 μm).

Fixing this monolayer with clean glass platelets finally results in imprints with the highest possible interaction area. As can be seen from Figures 3a and b, incorporated cells can ‘lie’ in the polymer surface, which not only facilitates the inclusion but also enhances the adhesion in the layer. In this way, the frequency response can be further improved. The left picture in Figure 3 was recorded with an Atomic Force Microscope, which represents an important tool in surface characterization. Apart from that, also reflected-light microscopy can be used for characterizing the respective surface (Figure 3).

**Figure 4.**
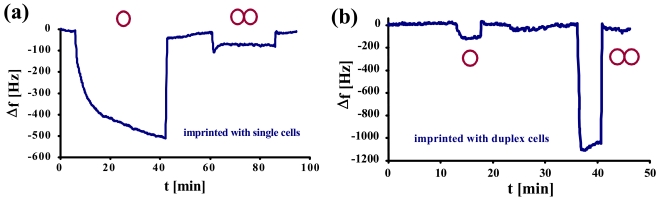
QCM responses of MIP exposed to single and duplex cells (1.17 × 10^6^ cells/μL).

Studies on selectivity of such MIPs on mass-sensitive sensors were compared to QCMs imprinted with single yeast cells. In Figure 4a, a normal yeast-sensitive sensor was exposed to a solution of single cells and to synchronized duplex cells of the same concentration (1.17 × 10^6^ cells/μL). The same measurement was repeated with a sensor for duplex cells (Figure 4b).

Here, it becomes evident that the two sensors show contrary behavior. While the frequency of the normal yeast sensor declines only by ∼100 Hz, the one for duplex cells shows a frequency drop of more than 1 kHz. Generally, both the sensors imprinted with single yeast cells (a) and with duplex cells (b), respectively, exhibit low cross-sensitivity and, at the same time, strong affinity to their own template. This can also be seen by the different response times between the template and the respective “competing” compound: the former usually shows (somewhat) longer response times. A reason for this may be that the cells have to arrange themselves in a way to optimally fit into the cavities. As selectivity in this case is strongly determined by geometry, nonspecific interactions are much faster.

**Figure 5.**
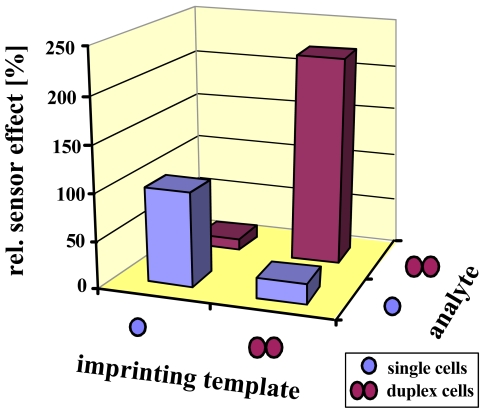
Normalized selectivity pattern (reference: single cell) of single/duplex yeast MIP.

The selectivity results are summarized in Figure 5. Again, both sensors prefer their respective template by at least a factor of five. Since the size of a duplex is less than twice the diameter of a single cell, this prevents incorporation of the incorrect analyte, being of course a main reason for selectivity. Additionally, the polysaccharide pattern of the cell wall slightly changes during the cell cycle, also leading to minor effects.

**Figure 6.**
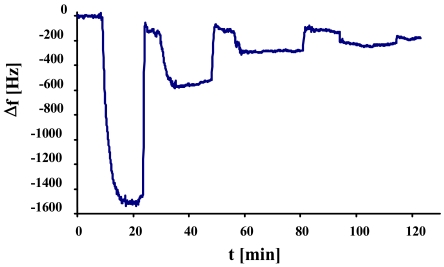
QCM sensor characteristic of duplex cell MIP: 2.3 × 10^5^, 1.15 × 10^5^, 5.8 × 10^4^ and 2.9 × 10^4^ cells/μL, respectively.

In the next step, we studied the sensor characteristics of duplex cell MIP, the result of which can be seen in Figure 6. The measurement shows linear behavior at low concentrations, whereas the effect at 2.3 × 10^5^ cells/μL exceeds the expected value probably due to multilayer adsorption of yeast on the MIP.

### 2.2. Artificial Yeast Master

**Figure 7.**
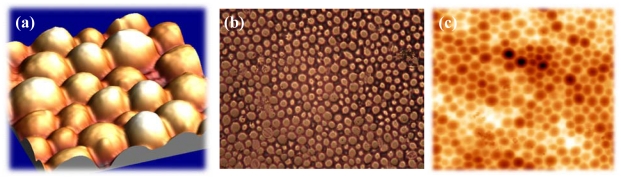
AFM image of an artificial yeast stamp (25 × 25 μm, vertical axis 5 μm) (a) and reflected-light microscopy (b) (125 × 100 μm). (c) AFM image MIP polyurethane resulting from artificial stamp.

As mentioned previously, we also focused on standardizing yeast MIPs via artificial stamps. For this purpose, an imprinted polyurethane layer is cast with another polymer (e.g., PDMS), thereby yielding plastic yeast cell copies. These are in turn used for templating in the imprinting procedure. Figure 7a shows an AFM image of such an artificial stamp, while Figure 7b depicts the same stamp in light microscope. Cavities produced by imprinting with a master stamp are presented in Figure 7c. The size of the cavities is the same as can be obtained by templating with the natural cells.

**Figure 8.**
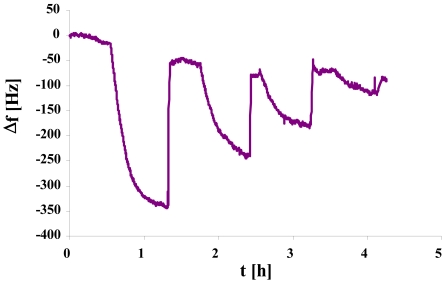
QCM sensor response of artificial stamp MIP exposed yeast at 3 × 10^5^, 1.5 × 10^5^, 8 × 10^4^ and 9 × 10^4^ cells/μL, respectively.

In the next step, we evaluated the sensor properties of the resulting imprints. Previous studies already proved the selectivity of such MIP between *S. cerevisiae* and *S. bayanus* [10]. Here, the cross-sensitivity towards the non-template is very low in spite of the similar geometry of the two analytes. The present work is targeted at the sensor characteristic, as can be seen in Figure 8 showing the individual frequency shifts of a QCM coated with stamp-templated yeast MIP at four concentrations.

**Figure 9.**
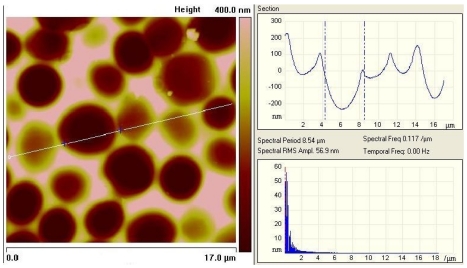
AFM image and section analysis of a polyurethane MIP of an artificial stamp mimicking growing yeast cells showing grown species and some buds.

Further work in this field concentrates on artificial stamps for different growth stages of *S. cerevisiae*. In recent studies, we succeeded in designing PDMS stamps from different yeast cell stages. For this purpose, we cultured yeast in YPD nutrient solution at room temperature. After approximately 20 minutes, the cells have reached early synthesis phase, with increased cell size and starting growth of buds. Figure 9 shows an AFM image of the PU-layer sensitive to cells of this growth stage generated by artificial stamp. Evidently, also these artificial polymer templates lead to cavities correctly reproducing the geometrical features of the budding yeasts.

**Figure 10.**
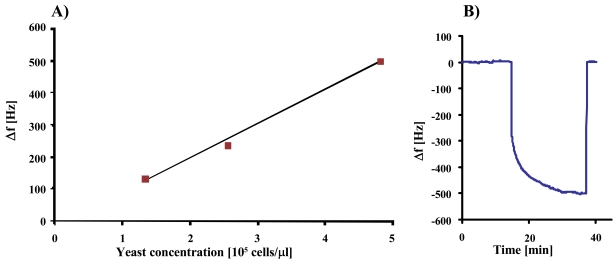
A) Sensor characteristic of S-phase yeast MIP resulting from artificial stamp the size of the squares indicates the error. B) QCM sensor response at 5 × 10^5^ cell/μL.

Figure 10 complements this picture by the sensor characteristic and an individual sensor response of the respective MIP material indicating also optimized interaction networks between polymer and analyte.

### 2.3. Multichannel Quartz Crystal Microbalances

**Figure 11.**
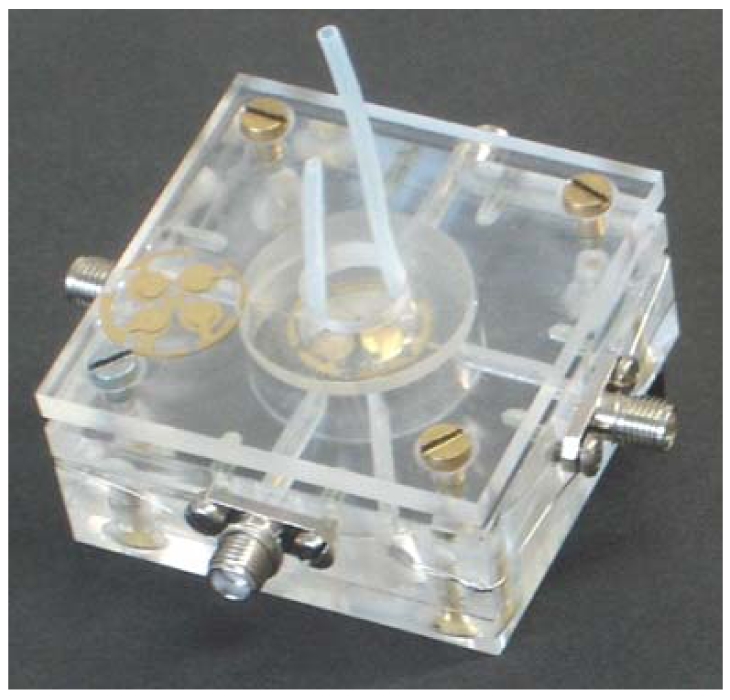
Measuring cell for a MQCM with 4 electrodes adapted for liquid phase application. Each individual channel is connected to its own oscillator.

Multichannel quartz crystal microbalances (MQCM) represent a promising approach for biosensor applications and have also attracted substantial interest in biotechnology and industry. Miniaturization of the devices results in substantial cost reduction and time saving. Besides, a multichannel sensor on a single quartz crystal improves the reproducibility and enables the simultaneous detection of several analytes. In this work, we designed QCM with four-electrode geometry and optimized them for measuring in liquid phase. In order to minimize the frequency interference between the individual channels, the size and thickness of the electrodes had to be carefully chosen. The final structures are 3.5 mm in diameter on the side facing the analyte chamber and 3 mm on the back side. The thickness of the gold layer is 120–140 nm. The measuring cell for a 4-channel sensor is shown in Figure 11.

One of the main problems in using multi-electrode setups on QCM in liquid phase is possible cross talk between the channels leading to impaired results. Therefore, neither the individual resonance frequencies, nor the electrodes should be too close together. The former is usually already given by manual screen printing, which is a directional process and thus usually leads to certain variation in electrode thickness. For the latter, the diameter of the electrode pairs has to be optimized. According to our experience, the distance between neighboring electrode pairs should be in the range of two millimeters. Substantially decreasing the electrode diameters, however, leads to reduced sensitivity. Therefore, the abovementioned dimensions are the result of longer lasting optimization.

**Figure 12.**
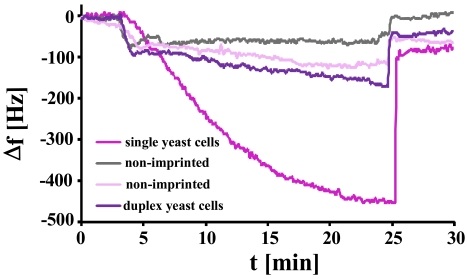
10 MHz MQCM with four electrodes exposed to a solution of single yeast cells (0.5 mg/mL ≈ 1.15 × 10^5^ cells/μL). The two non-imprinted electrodes as well as the one imprinted with synchronized duplex cells show only a minor frequency decrease, while the MIP imprinted with single cells responds strongly.

For the first tests, we coated the MQCM with polyurethane and imprinted them with different growth stages of *S. cerevisiae*. One electrode is imprinted with single cells and another with synchronized duplex cells, while the two remaining electrodes are left non-imprinted. Each channel is now connected with a dedicated oscillator and read out with a frequency counter and custom-made software. The resulting measurement in Figure 12 shows the sensor response to the addition of yeast (5 mg/mL). Here, the frequency decrease of the yeast imprinted electrode (single cells) obviously outperforms the others by about 400 Hz. The electrode imprinted with duplex cells shows a slightly higher effect than the non-imprinted ones which reflect the unspecific interactions of the analyte with the polymer. Compared to the previous measurements, the sensor responses are higher in this case, which is due to differences in thickness of the quartz substrate and electrode geometry, respectively.

## 3. Experimental Section

### 3.1. General

All chemicals were purchased in highest available purity and used as received. Baker's yeast was obtained as obtainable in trade and purified with distilled water. For mass-sensitive measurements, 10 MHz QCMs were custom-made by screen-printing the desired electrode structures onto commercially available AT-cut quartz blanks with 168 μm thickness and 15.5 mm diameter. Organic residues were removed while burning at 400°C for two hours. As the frequency determining elements, these sensors were connected to home-made oscillating circuits and read out with an Agilent 53131A frequency counter and the corresponding software.

### 3.2. Yeast Imprinting

Yeast-sensitive sensors were generated by molecular imprinting of polyurethane films on 10 MHz quartz plates. For such layers, the polymer mixture comprised of 40.2% w/w 4,4′-diisocyanato diphenylmethane, 47.8% w/w bisphenol A and 12% w/w phloroglucinol was dissolved in tetrahydrofuran and stirred at 70 °C until the gel point was reached. This pre-polymer was then spin coated on quartz blanks at 2,500 rpm to achieve flat and homogenous layers. For surface imprinting with single yeast cells, purified *Saccharomyces cerevisiae* was applied on glass slides and fixed on the coated QCMs. After complete polymerization over night, the stamps and templates were removed with water in an ultrasonic bath.

Duplex cells were obtained by synchronization of *S. cerevisiae* with *N*-hydroxyurea due to inhibition of mitosis [11]. The cell culture was grown at room temperature in a YPD nutrient solution made of 3 mg/mL yeast extract, 3 mg/mL malt extract, 5 mg/mL peptone and 10 mg/mL glucose in water. The culture medium containing 0.76 g of the cytotoxin was stirred for approximately five hours until about 95% of the cells were stopped at the end of the synthesis phase of the cell cycle before the onset of cell division. The duplex cells were separated from the growing medium by washing with distilled water and subsequent centrifugation for three times. For surface imprinting with synchronized yeast, PU was again spin cast on the quartz platelets. The concentrated duplex cells were then dropped onto the electrode to be printed and again spun at 2,500 rpm, thereby producing only a monolayer of duplex cells. These were fixed in horizontal position with clean glass stamps and removed after polymerization was completed over night.

### 3.3. Plastic Master Stamps

For artificial yeast stamps, we applied polydimethylsiloxane (PDMS) to generate hydrophobic master stamps. In the first step, small glass stamps were coated with polyurethane and the surface was structured via imprinting with different growth stages of yeast by means of the respective strategy described above. PDMS is based on the Sylgard 184 Silicone elastomer kit, which facilitates the preparation and optimization of composition of the silicone. The desired rigidity can easily be achieved by variation of the ratio of mixture. Here, base material and hardening agent were mixed at a rate of 10:1 and freed from gas in an exsiccator. The prepared imprinted glass slides were then arranged in a dish and then covered with the silicone mixture. The covered dish was left to harden for two days at room temperature, before the plastic yeast cells were cut out and detached from the PU-layer. The master stamps were then used to generate MIPs on 10 MHz quartz microbalances by applying the same imprinting technique as with native cells.

## 4. Conclusions

Selective detection of yeast and several of its growth stages can be achieved by imprinting techniques. Measurements with 10 MHz QCMs imprinted with synchronized yeast cells demonstrated the capability of such sensors to distinguish between duplex and single yeast cells, indicating that different growth stages can be distinguished by the sensors, which is potentially interesting for monitoring fermentation processes. Moreover, we produced artificial plastic copies of natural yeast cells. Molecular imprinting with such master stamps mimicking native yeast cells in G_0_ and early S phase leads to structured polymer surfaces selectively incorporating the respective template growth stage of the cell. In addition to the advantage of improved reproducibility and standardization, such layers on mass-sensitive devices feature the same selectivity and sensitivity as MIPs generated using native cells. Hence, substitution of the natural template with the artificial analogue represents substantial advancement in bioanalyte sensing.

Another promising approach for enhancing sensor systems is based on multichannel quartz crystal microbalances (MQCM). A one-chip system comprising a 4-electrode design was developed and optimized for the measurement in liquid phase. In order to study the sensor characteristics, the MQCM was imprinted with one example growth stage of *S. cerevisiae*. Together with the selectivity data obtained from the dual electrode systems, this opens up the way to differentiate between the individual stages of development. Since such sensors already tested in long-run analyses allow the supervision of four analytes at the same time, corresponding analytical systems open up new opportunities for the application in biotechnology and process control.
